# A rare renal neoplasm: Case report of mucinous tubular and spindle cell carcinoma

**DOI:** 10.1016/j.radcr.2026.01.023

**Published:** 2026-02-06

**Authors:** Salma El Aouadi, Soukaina Allioui, Soukaina Bahha, Ouiam Taibi, Fatima Zahra Laamrani, Youssef Omor, Rachida Latib, Sanae Amalik

**Affiliations:** Department of Radiology, National Institute of Oncology, Ibn Sina University Hospital Center, Rabat, Morocco

**Keywords:** Mucinous tubular and spindle cell carcinoma, Kidney tumor, Magnetic resonance imaging, Histopathology

## Abstract

Mucinous tubular and spindle cell renal cell carcinoma (MTSRCC) is a rare subtype of renal cell carcinoma with distinctive histological features and generally favorable prognosis. We report the case of a 52-year-old woman who presented with right lumbar pain. CT and MRI revealed a well-defined solid renal mass with mild, homogeneous enhancement. The patient underwent tumorectomy, and histological examination confirmed mucinous tubular and spindle cell renal cell carcinoma. The favorable postoperative outcome in this case illustrates both the diagnostic value of imaging and histology and the good prognosis associated with MTSRCC.

## Introduction

Mucinous tubular and spindle cell renal cell carcinoma (MTSRCC) is an uncommon variant of renal cell carcinoma (RCC) [1]. Histologically, it is composed of cuboidal cells arranged in tubular formations, admixed with spindle cell areas, within a variably abundant mucinous stroma [1]. This neoplasm was formally classified as a distinct renal tumor entity in the 2004 World Health Organization classification [2]. Compared with other malignant renal neoplasms, MTSRCC is generally associated with a more favorable prognosis [2].

Although MTSRCC has been previously described in the literature, most published studies have mainly focused on histopathological features, with limited emphasis on detailed clinico-radiological correlation. This report contributes an additional well-documented case highlighting imaging findings and their correlation with pathological features, thereby addressing an aspect that remains insufficiently detailed in previously published studies.

## Case report

A 52-year-old woman with no relevant past medical history, presented with right-sided lumbar pain persisting for 2 months. Physical examination was unremarkable, with normal vital signs, and no palpable abdominal mass. Laboratory investigations, including complete blood count, renal and liver function tests, serum creatinine, and urinalysis, were all within normal limits, indicating preserved renal function and absence of systemic inflammation.

**Fig. 1:**
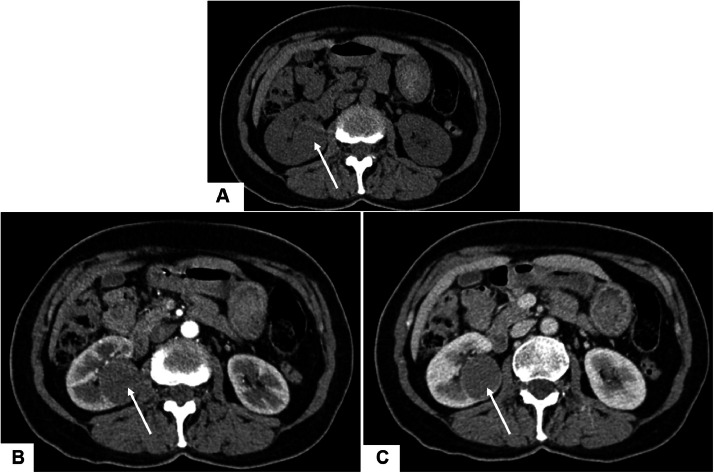
Axial contrast-enhanced CT images of the abdomen: (A) non-contrast, (B) arterial phase, and (C) portal venous phase. A well-circumscribed ovoid mass (arrow) is identified at the lower pole of the right kidney, demonstrating a mean attenuation of approximately 33HU on non-contrast images, 48 HU in the arterial phase, and 53 HU in the portal venous phase, consistent with mild and homogeneous enhancement.

**Fig. 2:**
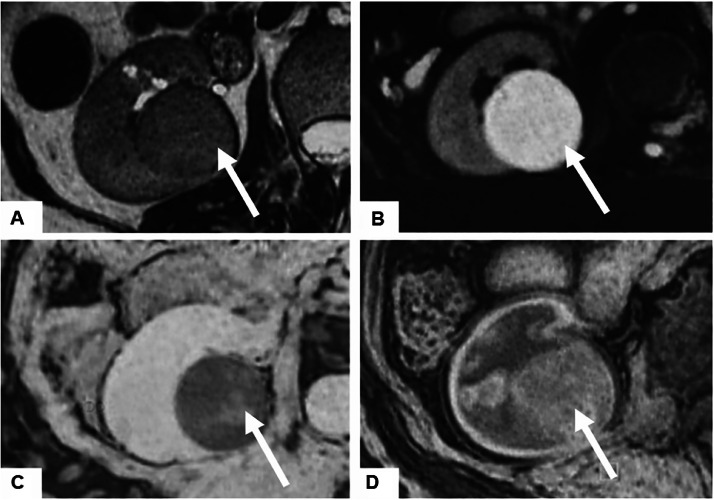
Axial T2-weighted MR image (A), axial diffusion-weighted image (b = 800 sec/mm²) (B), axial ADC map (C), and axial T1-weighted fat-saturated image after gadolinium injection (D): MRI showing a right renal mass (arrow) appearing isointense on T2-weighted images, with restricted diffusion, low ADC values, and homogeneous enhancement after gadolinium administration.

Given the persistence of symptoms, a contrast-enhanced abdominal computed tomography (CT) scan (Fig. 1) was performed. Imaging revealed a well-circumscribed, ovoid mass located at the lower pole of the right kidney, partially exophytic, measuring 34 × 29 mm. The lesion was isodense on non-contrast images and showed mild, homogeneous enhancement in the portal venous phase, without calcifications, necrosis, or signs of vascular invasion. No lymphadenopathy or distant lesions were detected. For better characterization, renal magnetic resonance imaging (MRI) (Fig. 2) was performed. The mass appeared isointense on both T1- and T2-weighted sequences, with restricted diffusion and low ADC values. After gadolinium injection, it demonstrated faint but homogeneous enhancement.

Based on the preoperative imaging findings, several differential diagnoses were considered. The main consideration was a low-grade RCC, particularly papillary RCC, given the small size of the lesion, its well-circumscribed margins, and its mild, homogeneous enhancement. Chromophobe RCC was also considered due to the indolent appearance and lack of aggressive imaging features. A benign renal tumor such as oncocytoma was another possibility, although the absence of a central scar and the presence of diffusion restriction made this diagnosis less likely. Given the indeterminate nature of the renal mass on preoperative imaging, a right tumorectomy was performed.

**Fig. 3:**
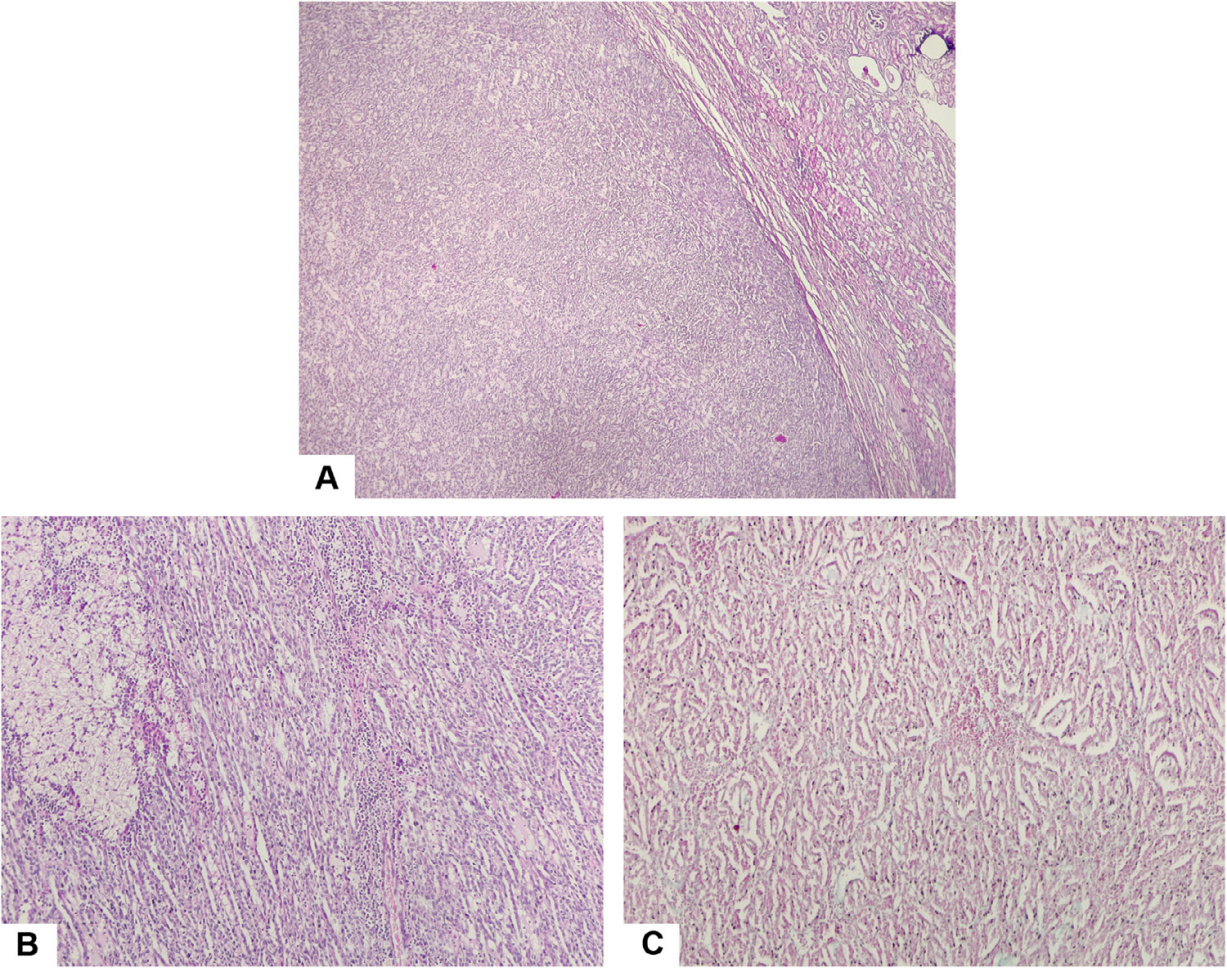
Histopathological images of the resected specimen at low magnification (A, B, C) : (A) Hematoxylin and eosin stain showing a well-circumscribed renal tumor composed of compact tubules and spindle-shaped cells. (B) Hematoxylin and eosin stain demonstrating elongated compact tubules interspersed with foamy histiocytes. (C) Alcian blue stain highlighting a myxoid stromal component.

Histopathological analysis of the resected specimen (Fig. 3) revealed a well-circumscribed carcinomatous proliferation composed of tubular structures arranged in parallel, admixed with fascicles of spindle cells. The tumor displayed low-grade cytological atypia, without sarcomatoid features. These findings were consistent with a MTSRCC, with negative surgical margins.

**Fig. 4:**
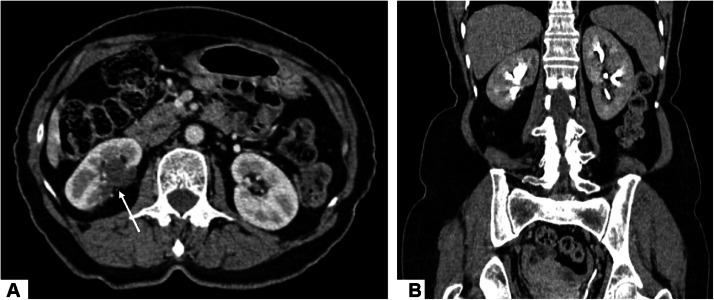
One-year follow-up contrast-enhanced CT: axial image in the portal phase (A) and coronal image in the delayed phase (B), showing a normal postoperative appearance of the right renal tumorectomy site (arrow). The renal parenchyma enhances homogeneously, with no residual lesion, and the kidney demonstrates normal contrast excretion.

The postoperative course was uneventful, with preserved renal function. At follow-up, the patient remained asymptomatic, and the 1-year control CT scans showed no signs of recurrence or distant metastasis (Fig. 4).

## Discussion

MTSRCC is an uncommon subtype of RCC [1]. It was first described in 1997 and was later recognized as a distinct entity in the 2004 World Health Organization (WHO) classification of renal tumors [2]. To date, fewer than 100 cases have been documented in the English-language literature [1].

MTSRCC occurs mainly in adults, with reported ages ranging from 13 to 82 years, and demonstrates a clear female predominance, with an approximate male-to-female ratio of 1:4 [3]. While certain patients may present with clinical symptoms such as flank pain, abdominal mass, or hematuria [4], most cases are detected incidentally during imaging examinations performed for unrelated indications [5]. Occasional associations have also been described with nephrolithiasis [1] and with tumors developing in the context of end-stage renal disease.

Radiologically, MTSRCC exhibits imaging features that differ from clear-cell RCC but resemble those of papillary RCC [6]. On CT, it generally appears as a well-defined, exophytic or endophytic mass with an expansile, round or ovoid contour, showing iso- to slightly hypoattenuating density [1]. MRI typically demonstrates mild hypointensity with focal hyperintensity on T2-weighted images, restricted diffusion on DWI, and a pattern of slow, progressive enhancement often plateauing in the delayed phases [7].

The main radiological differential diagnoses of MTSRCC include clear-cell, papillary, and chromophobe RCC [6]. On contrast-enhanced CT, clear-cell RCC typically shows marked heterogeneous enhancement, whereas papillary RCC demonstrates low, homogeneous enhancement [6]. MTSRCC usually exhibits mild and homogeneous enhancement, closer to papillary RCC [6]. On MRI, clear-cell RCC is often hyperintense on T2-weighted images, while papillary RCC is classically hypointense [6]. MTSRCC generally shows low to intermediate T2 signal intensity with diffusion restriction, overlapping with papillary RCC, which explains the preoperative diagnostic challenge [6].

The definitive diagnosis of MTSRCC relies on histopathological examination. Macroscopically, it usually presents as a well-circumscribed cortical mass, with a glistening mucoid cut surface of homogeneous color and firm consistency [8]. Microscopically, it is composed of parallel, tightly packed tubules and spindle cells separated by variable amounts of mucinous stroma [8]. Transitions between tubular and spindle components are frequently observed [8]. Reported histological variants include mucin-poor forms, spindle cell–dominant patterns, and tumors with high nuclear grade [8]. Necrosis and mitotic figures are rarely encountered [8].

The diagnosis of MTSRCC may be challenging because of its close histological resemblance to papillary RCC [7]. Immunohistochemistry, particularly the lack of CD10 expression, can aid in distinguishing between the 2 entities [9]. The differential diagnosis also includes sarcomatoid RCC and various mesenchymal tumors such as leiomyoma, angiomyolipoma, and inflammatory myofibroblastic tumor, which can usually be separated from MTSRCC based on their characteristic histological, immunohistochemical, and molecular profiles [7].

MTSRCC with classic morphology generally carries a good prognosis, and surgical excision—either partial or radical nephrectomy—is usually curative [1]. Most tumors are diagnosed at an early pathological stage [1]. Recurrence and metastasis are rare, but have been described, particularly in cases with high-grade nuclei or sarcomatoid change [1]. Nonetheless, distant spread has occasionally been reported even in tumors with typical low-grade morphology [10,11], underscoring the need for careful follow-up. There are currently no established systemic treatment guidelines for advanced disease, although isolated reports suggest potential benefit from targeted therapy [1].

This case adds to the limited number of reported MTSRCCs and further supports the generally favorable prognosis associated with this rare entity.

## Conclusion

This case illustrates the importance of considering MTSRCC in the differential diagnosis of renal masses. Radiological evaluation combined with histopathology is essential for accurate diagnosis. Surgical excision, either by tumorectomy or partial nephrectomy, remains an effective therapeutic option, and prognosis is generally favorable when complete resection is achieved.

## Author’s contributions

All authors contributed to this work. All authors have read and approved the final version of the manuscript.

## Ethics approval

Our institution does not require ethical approval for reporting individual cases or case series.

## Patient consent

Written informed consent was obtained from the patient for their anonymized information to be published in this article.
